# Models for skin tumour risks in workers exposed to mineral oils.

**DOI:** 10.1038/bjc.1990.435

**Published:** 1990-12

**Authors:** B. Järvholm, D. Easton

**Affiliations:** Department of Occupational Medicine, Sahlgren University Hospital, Göteborg, Sweden.

## Abstract

The relationship between skin tumours in man and exposure to polyaromatic hydrocarbons has been studied in lathe operators exposed to cutting oils. Seven cases of scrotal cancer and 13 cases of senile keratosis and keratoacanthoma were observed. The risk varied as the 1.6th power of duration of exposure for cancer on the scrotum and the 2.4th power for tumours on the hand and forearms. These results accord well with experiments on animals. There was some evidence of an increasing trend in risk with increasing age at first exposure.


*Br. J. Cancer* (1990), **62**, 1039–1041 © Macmillan Press Ltd., 1990

### Models for skin tumour risks in workers exposed to mineral oils

B. Järvholm[1] & D. Easton[2]

[1]*Department of Occupational Medicine, Sahlgren University Hospital, S:t Sigfridsgatan 85, Göteborg, Sweden; and* [2]*Section of Epidemiology, Institute of Cancer Research, Royal Cancer Hospital, Sutton, Surrey SM2 5NG, UK.*

**Summary** The relationship between skin tumours in man and exposure to polyaromatic hydrocarbons has been studied in lathe operators exposed to cutting oils. Seven cases of scrotal cancer and 13 cases of senile keratosis and keratoacanthoma were observed. The risk varied as the 1.6th power of duration of exposure for cancer on the scrotum and the 2.4th power for tumours on the hand and forearms. These results accord well with experiments on animals. There was some evidence of an increasing trend in risk with increasing age at first exposure.

Exposure to some mineral oils is a well known cause of skin tumours in both laboratory animals and man (IARC, 1984). The carcinogenic properties of the oils is thought to be mainly due to their content of polycyclic aromatic hydrocarbons (IARC, 1984).

A common animal model for carcinogenesis involves painting polycyclic aromatic hydrocarbons (PAH) or mineral oils containing PAH on the skin, and several experiments studying dose–response relationship in this animal model have been reported. However, there are few data on the relationship between exposure to PAH and skin tumours in humans. Case reports have indicated that several years typically elapse between onset of exposure and the occurrence of tumours (Cruickshank & Squire, 1950; Cruickshank & Gourevitch, 1952; Mastromatteo, 1955; Waldron, 1983; Waldron *et al*., 1984). The populations on which these case reports are based are, however, not described in such a way that risk estimates can be calculated.

We have previously reported an increased risk of squamous cell carcinomas on the skin of scrotum and premalignant skin lesions on the hand and forearms in workers exposed to cutting oils (Järvholm *et al*., 1985; Järvholm & Lavenius, 1987). We here report analyses of the relationship between these tumours and duration of exposure to mineral oils.

### Subjects and methods

### *Subjects*

The study is based on men employed as lathe operators at an industry producing bearing rings located in Göteborg, Sweden. Because of different methods of ascertaining scrotal cancers and tumours on the hands and forearms, slightly different (though overlapping) cohorts were studied for the two sites.

The 'scrotal cancer' cohort (I) consists of men with at least 5 years service as a lathe operator in certain departments, a total of 251 men. The 'skin tumour' cohort (II) consists of lathe operators in certain departments who were screened for skin tumours some time between 1960 and 1980 and had at least 5 years of service as a lathe operator in these departments, a total of 294 men. The selection procedures have earlier been described in detail (Järvholm *et al*., 1985; Järvholm & Lavenius, 1987).

### *Methods*

The occurrence of skin cancer on scrotum between 1958 and 1983 in cohort I was collected through a linkage with the Swedish Cancer Register. In total there were seven cases.

The occurrence of primary tumours on the skin of forearms and hands in cohort II were found by scrutinising medical files (Järvholm *et al*., 1985). In total there were five cases of senile keratosis and eight cases of keratoacanthoma. All tumours were verified by microscopical analysis of biopsies. No distinction has been made between these two types of skin tumours in the analysis.

The incidence of skin cancer on the scrotum in Sweden is about 0.5 per $10^6$ per year, i.e. about two cases a year (Cancer Incidence in Sweden, 1985). Premalignant skin lesions and kerato-acanthomas on the hands and forearms also seem to be rather rare. Järvholm *et al*. (1985) have estimated the incidence to be about three per $10^5$ per year in males 20–69 years of age. This estimate was based on biopsied lesions and included too few cases to allow a stratification into different age-classes. Even allowing for the uncertainty in such estimates, therefore, it is reasonable to assume that all the studied tumours were caused by occupational exposure to mineral oils. (A crude estimate of the total numbers of scrotal skin cancer and premalignant skin lesions on the hands and forearms was about 0.1 cases in the cohorts.)

The cutting oils used in these departments were of similar composition until 1975, based on acid refined mineral oils (at least 90%) with some sulphur (1% or less). In 1975 the acid refined mineral oils were replaced by solvent refined mineral oils. Animal experiments and chemical analyses indicate that solvent refined mineral oils do not constitute a risk for skin tumours (IARC, 1984); we have therefore ignored exposure occurring after 1975. Although it is very difficult to estimate the skin exposure in occupational groups, earlier studies have indicated that the exposure to hands and forearms was similar between 1960 and early 1970s (Järvholm *et al*., 1985).

### *Statistical methods*

Since the background risk of both scrotal and the other skin tumours is low, all analyses are in terms of absolute risk (AR), namely the number of cases per person-years of observation. The principal interest was to determine whether the cancer risk varied as a power function of duration of exposure, as suggested by the animal experiments and predicted by a simple multistage model of carcinogenesis, i.e.:

$$\log(\mathrm{AR}) = a + b \times \log \text{ (duration of exposure)}$$

The other questions of interest were to determine whether, in addition to the effect of duration of exposure, the risk varied with age at onset of exposure or with time after stopping exposure.

The various models were fitted using the package GLIM (Baker & Nelder, 1978), assuming that the observed number of cells followed a Poisson distribution with mean equal to the predicted number under the given model. For testing the significance of adding age at first exposure and/or time after stopping exposure the likelihood ratio test was used.

Correspondence: B. Järvholm.
Received 24 October 1989; and in revised form 7 February 1990.

### Results

Table I and Figure 1 show the risk of skin cancer on the scrotum and skin tumours on the hands and forearms in relation to duration of exposure. Both relationships are well described by power laws with exponents 1.6 for scrotal cancer (standard error = 0.8, $P = 0.02$) and 2.4 for skin tumours on the hands and forearms (standard error = 0.7, $P = 0.0003$). Both sites are in fact consistent with power laws with the same exponent; by requiring the same relationship between AR and duration of exposure we obtained an estimate for this exponent of 2.2 (s.e. = 0.6, $P = 0.0001$).

There is some suggestion of an increasing risk for skin tumours with increasing age at onset of exposure (Table II). After allowing for duration of exposure, the effect of age at onset of exposure was significant for skin cancer on the scrotum ($\chi^2$ for trend = 5.7, $P = 0.02$), and marginally so for skin tumours on the hands and forearms ($\chi^2$ for trend = 2.8, $P = 0.1$). If age at first exposure is included in the model, the exponents for the relationship between risk and duration of exposure are altered to 2.3 for cancer on the scrotum and 2.8 for tumours on hands and forearms.

Adding time since onset of exposure did not improve the fit of the model ($\chi^2 = 0.1$ and $\chi^2 = 0.7$ respectively).

There was no evidence of an increased risk after stopping exposure ($\chi^2 = 0.2$ and $\chi^2 = 1.0$ respectively for the two sites for adding time since stopping exposure to the model), and in fact the risk was lower after exposure than during exposure for both cohorts, though not significantly. It should be noted, however, that data for examining the risk after exposure had ceased was extremely limited in both cohorts, especially in cohort II.

### Discussion

The results indicate that tumours on the human skin caused by PAH follow a similar relationship to duration of exposure as in animal experiments, namely a power function with an exponent of two or three (Lee & O'Neill, 1971; Peto *et al.*, 1975). This is the type of relationship predicted by a simple multistage model (Armitage & Doll, 1961). It should be borne in mind that the exposure of these humans is likely to be far less homogeneous than exposure in experiments on animals. The exposure may vary between individuals and during time in the same individual. Nevertheless, the production in these departments has been quite stable during the years and also the type of cutting oils. The concentration of PAH may have varied as crude oils from different areas may have different composition of PAH (Medical Research Council, 1968) and the acid refining process may have varied. Used oils seem to be more carcinogenic (Thony *et al.*, 1976) than new oils but there was rarely any instantaneous replacements of the total bulk of oils. The losses during production were replaced continuously.

The analysis suggests that the age at onset of exposure may have influenced the risk with a higher risk in men with onset of exposure at an older age. This is somewhat in variance with experiments on animals which indicates that the risk of skin tumours caused by PAH is largely independent of age (Peto *et al.*, 1975). If this effect were genuine it would perhaps indicate that these tumours were initiated by a mechanism unrelated to mineral oils and that the mineral oils acted at a later stage in the carcinogenic process in humans. It should be noted, however, that we have only been able to register exposure in this industry. Some of the men may have been exposed to mineral oils in other industries before they were employed in this factory. This would mean an underestimation of duration of exposure, especially for men with old age at onset of exposure and lead to an artefactual effect of age at first exposure.

Duration of exposure is of course strongly correlated to age. An analysis for scrotal skin cancer indicated that a power-function of age was a slightly better predictor of AR than duration of exposure (−2 likelihood = 51.6 compared to 52.6). On the other hand for the premalignant lesions duration of exposure fitted the data slightly better than age (−2 likelihood = 41.5 compared to 44.5). The best fitting power functions for age had an exponent of 5.6 for both sites. If the risk was simply a function of age rather than duration, then the risk would continue to rise after exposure ceased, whereas the duration model would predict a constant risk. There was no evidence that the risk of scrotal skin cancer did rise after ceasing exposure, and some weak

**Figure 1** Relationship between duration of exposure and absolute risk for skin cancers on the scrotum (×) and skin tumours on the hand and forearms (O). The lines are the fitted log–log models (see text). Scrotum: AR = 12.25 × $10^{-6}$ × (duration)$^{1.60}$. Hands and forearms: AR = 3.92 × $10^{-6}$ × (duration)$^{2.42}$.

**Table II** Absolute risk (AR) per 1,000 person-years of skin tumours by age at onset of exposure and fitted risks (FR) after adjusting for duration of exposure

| *Age at onset of exposure* | *Scrotal cancer* | | | *Skin tumours on the hands and forearms* | | |
|---|---|---|---|---|---|---|
| | *No. of cases* | *AR* | *FR*[a] | *No. of cases* | *AR* | *FR*[a] |
| ⩽24 | 0 | 0 | 0.00 | 2 | 2.6 | 0.19 |
| 25–29 | 2 | 1.4 | 0.51 | 7 | 9.6 | 1.06 |
| ⩾30 | 5 | 2.6 | 1.00 | 4 | 4.8 | 1.00 |

[a]Risks after fitting duration of exposure, relative to the risk in men at least 30 years old at first exposure.

**Table I** Absolute risk (AR) of skin tumours per 1,000 person-year according to duration of exposure

| *Duration of exposure (years)* | *Scrotal cancer* | | | *Skin tumours on the hands and forearms* | | |
|---|---|---|---|---|---|---|
| | *Person-years* | *No. of cases* | *AR* | *Person-years* | *No. of cases* | *AR* |
| 5–14 | 2126.0 | 1 | 0.47 | 1175.2 | 1 | 0.85 |
| 15–24 | 1662.7 | 2 | 1.2 | 647.4 | 3 | 4.7 |
| 25–34 | 589.4 | 2 | 3.4 | 363.9 | 7 | 19.0 |
| ⩾35 | 379.5 | 2 | 5.3 | 113.5 | 2 | 18.0 |

evidence that the risk did not continue to rise with age, at the same rate ($\chi^2 = 2.0$ for adding time since last exposure to age in the model). For premalignant lesions on the hands and forearms there was no data available for studying the risk after stopping exposure. It is thus impossible to be certain from this data whether age *per se*, or duration of exposure is a better model. However, the fitted models using a power function of duration of exposure, including age at onset of exposure seem to explain all the observations.

Long-term exposure to these mineral oils constituted a significant risk for skin tumours. For example, the estimated cumulative risk after 30 years continuous exposure was 14% for skin tumours on the hands or forearms. The corresponding risk for skin cancer on the scrotum was 4%. The tumours do of course occur after exposure had ceased and consequently lifetime risk estimates would be greater than these figures. The new refining procedures, such as solvent refining, have probably eliminated this risk in cutting oils; solvent refining almost totally eliminate the PAH in the oil (IARC, 1984). The concentration of PAH may however increase during use. This does not seem to be a large problem in cutting oils (LaFontaine, 1978), but may be a risk in other operations where the oil is more severely heated, i.e. quenching.

The risk for skin tumours on the hands and forearms seems to be higher than the risk for skin tumours on the scrotum in men exposed to cutting oils. It seems reasonable that the risk for skin tumours on the hands and forearms is higher as these parts of the body are more exposed to the oils than the scrotum. While there are several reports about skin cancer on the scrotum due to such exposure few papers have been published about skin tumours on the hands or forearms. This discrepancy is probably due to observation bias. The tumours on hands and forearms are often observed in their premalignant stage and consequently rarely reported to cancer registries (Järvholm *et al*., 1985).

In summary this study supports the view that the risk of skin tumours caused by PAH varies as the 2nd or 3rd power of duration of exposure. Long-term heavy exposure to poorly refined mineral oils constitutes a substantial risk for squamous cell skin tumours.

This work was supported by the Swedish Work Environment Fund. The Institute of Cancer Research is supported by the Cancer Research Campaign and the Medical Research Council.

### References

ARMITAGE, P. & DOLL, R. (1961). Stochastic models for cancerogenesis. In *Proceedings of the Fourth Berkeley Symposium on Mathematical Statistics and Probability, Vol. 4*, Neyman, J. (ed.) p. 19. University of California Press: Berkeley.

BAKER, R.J. & NELDER, J.A. (1978). *Generalized Linear Interactive Modeling (GLIM) System. Release 3.* Numerical Algorithms Group: Oxford.

CANCER INCIDENCE IN SWEDEN (1958) [1959 . . . 1985]. National Board of Health and Welfare. The Cancer Registry: Stockholm.

CRUICKSHANK, C.N.D. & GOUREVITCH, A. (1952). Skin cancer on the hand and forearm. *Br. J. Indust. Med.*, **9**, 74.

CRUICKSHANK, C.N.D. & SQUIRE, J.R. (1950). Skin cancer in the engineering industry from the use of mineral oil. *Br. J. Indust. Med.*, **7**, 1.

IARC (1984). *IARC Monographs on the Evaluation of the Cancerogenic Risk of Chemicals to Humans. Polynuclear Aromatic Compounds, Part 2.* IARC, WHO: Lyon.

JÄRVHOLM, B., FAST, K., LAVENIUS, B. & 1 other (1985). Exposure to cutting oils and its relation to skin tumors and premalignant skin lesions on the hands and forearms. *Scand. J. Work. Environ. Health*, **11**, 365.

JÄRVHOLM, B. & LAVENIUS, B. (1987). Mortality and cancer morbidity in workers exposed to cutting fluids. *Arch. Environ. Health*, **42**, 361.

LAFONTAINE, M. (1978). *Huiles Minerales et Cancers Cutanes.* Institut National De Recherche et de Securite: Paris.

LEE, P.N. & O'NEILL, J.A. (1971). The effect both of time and dose applied on tumour incidence rate in benzopyrene skin painting experiments. *Br. J. Cancer*, **25**, 759.

MASTROMATTEO, E. (1955). Cutting oils and squamous-cell carcinoma. Part I. Incidence in a plant with a report of six cases. *Br. J. Indust. Med.*, **12**, 240.

MEDICAL RESEARCH COUNCIL (1968). *The Carcinogenic Action of Mineral Oils. A Chemical and Biological Study.* MRC: London.

PETO, R., ROE, F.J.C., LEE, P.N. & 3 others (1975). Cancer and aging in mice and men. *Br. J. Cancer*, **32**, 411.

THONY, C., THONY, J., LAFONTAINE, M. & 1 other (1976). Hydrocarbures polycycliques aromatiques cancerogenes dans les produits petroliers preventions possibles du cancer des huiles minerales. *Inserm. Symp. Ser.*, **52**, 165.

WALDRON, H.A. (1983). A brief history of scrotal cancer. *Br. J. Indust. Med.*, **40**, 390.

WALDRON, H.A., WATERHOUSE, J.A.H. & TESSEMA, N. (1984). Scrotal cancer in the West Midlands 1936–76. *Br. J. Indust. Med.*, **41**, 437.


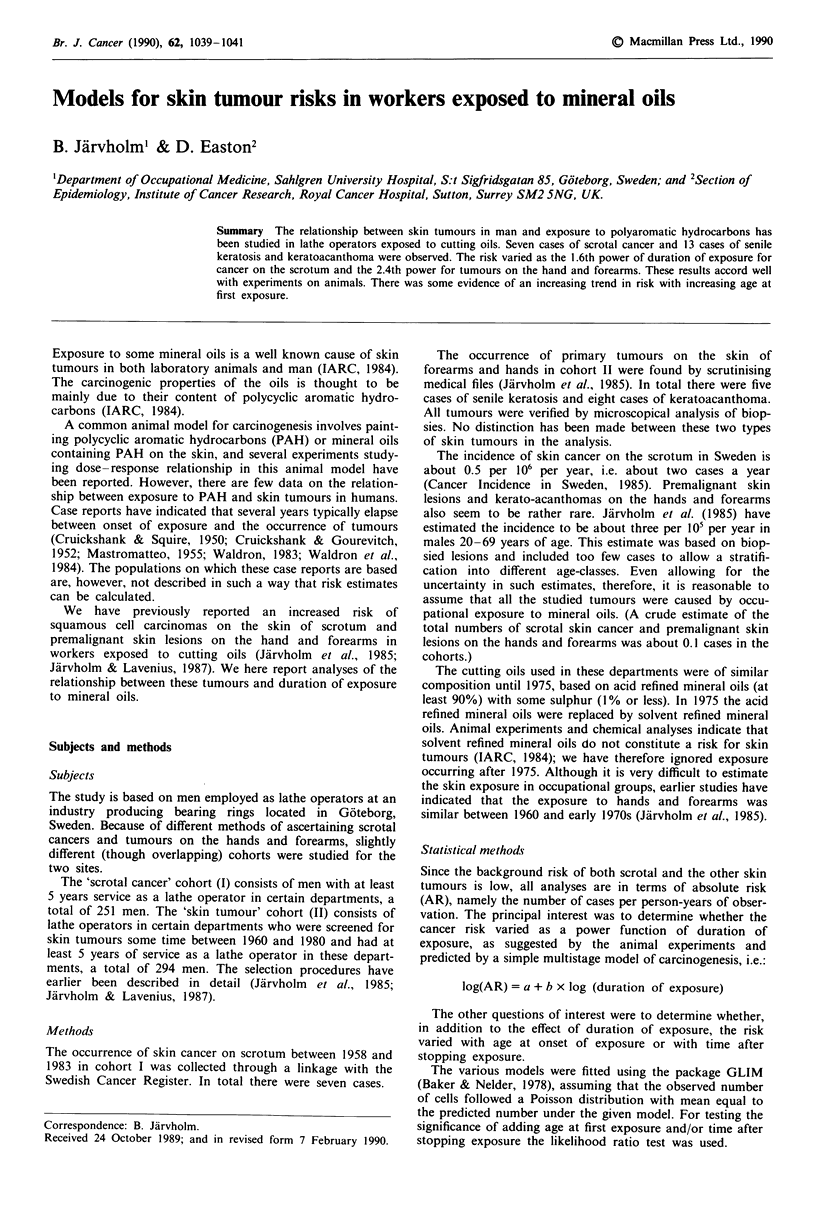

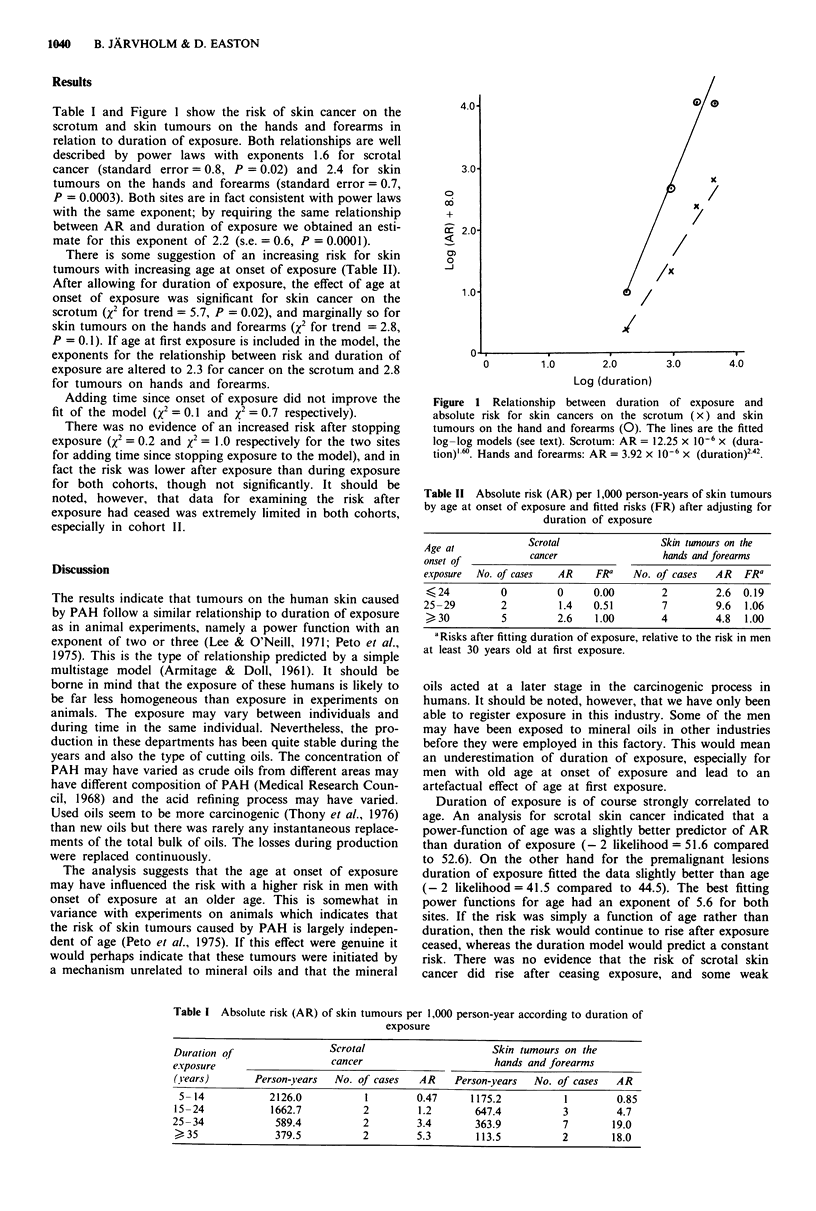

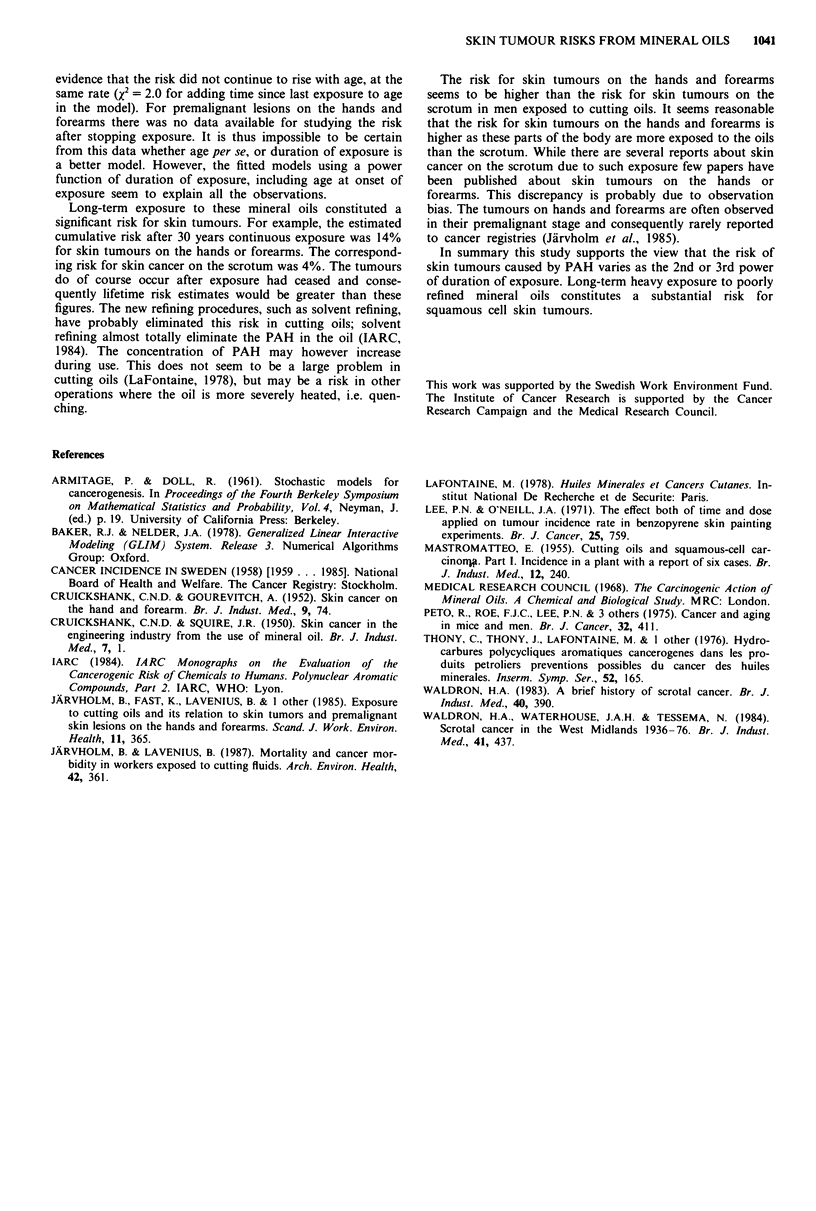


## References

[OCR_00359] CRUICKSHANK C. N. D., GOUREVITCH A. (1952). Skin cancer of the hand and forearm.. Br J Ind Med.

[OCR_00363] CRUICKSHANK C. N. D., SQUIRE J. R. (1950). Skin cancer in the engineering industry from the use of mineral oil.. Br J Ind Med.

[OCR_00373] Järvholm B., Fast K., Lavenius B., Tomsic P. (1985). Exposure to cutting oils and its relation to skin tumors and premalignant skin lesions on the hands and forearms.. Scand J Work Environ Health.

[OCR_00379] Järvholm B., Lavenius B. (1987). Mortality and cancer morbidity in workers exposed to cutting fluids.. Arch Environ Health.

[OCR_00388] Lee P. N., O'Neill J. A. (1971). The effect both of time and dose applied on tumour incidence rate in benzopyrene skin painting experiments.. Br J Cancer.

[OCR_00393] MASTROMATTEO E. (1955). Cutting oils and squamous-cell carcinoma. I. Incidence in a plant with a report of six cases.. Br J Ind Med.

[OCR_00401] Peto R., Roe F. J., Lee P. N., Levy L., Clack J. (1975). Cancer and ageing in mice and men.. Br J Cancer.

[OCR_00405] Thony C., Thony J., Lafontaine M., Limasset J. C. (1976). Hydrocarbures polycycliques aromatiques cancerogenes dans les produits pétroliers: préventions possibles du cancer des huiles minérales. IARC Sci Publ.

[OCR_00411] Waldron H. A. (1983). A brief history of scrotal cancer.. Br J Ind Med.

[OCR_00415] Waldron H. A., Waterhouse J. A., Tessema N. (1984). Scrotal cancer in the West Midlands 1936-76.. Br J Ind Med.

